# Hydroxyapatite Formation on Self-Assembling Peptides with Differing Secondary Structures and Their Selective Adsorption for Proteins

**DOI:** 10.3390/ijms20184650

**Published:** 2019-09-19

**Authors:** Suzuka Kojima, Hitomi Nakamura, Sungho Lee, Fukue Nagata, Katsuya Kato

**Affiliations:** National Institute of Advanced Industrial Science and Technology, 2266-98, Anagahora, Shimo-Shidami, Moriyama-ku, Nagoya, Aichi 463-8560, Japan; suzuka-kojima@aist.go.jp (S.K.); hi.nakamura@aist.go.jp (H.N.); sungho.lee@aist.go.jp (S.L.); f.nagata@aist.go.jp (F.N.)

**Keywords:** solid-phase peptide synthesis, hydroxyapatite, peptide, secondary structure, selective protein adsorption, biotemplate

## Abstract

Self-assembling peptides have been employed as biotemplates for biomineralization, as the morphologies and sizes of the inorganic materials can be easily controlled. We synthesized two types of highly ordered self-assembling peptides with different secondary structures and investigated the effects of secondary structures on hydroxyapatite (HAp) biomineralization of peptide templates. All as-synthesized HAp-peptides have a selective protein adsorption capacity for basic protein (e.g., cytochrome c and lysozyme). Moreover, the selectivity was improved as peptide amounts increased. In particular, peptide–HAp templated on β-sheet peptides adsorbed more cytochrome c than peptide–HAp with α-helix structures, due to the greater than 2-times carboxyl group density at their surfaces. It can be expected that self-assembled peptide-templated HAp may be used as carriers for protein immobilization in biosensing and bioseparation applications and as enzyme-stabilizing agents.

## 1. Introduction

As represented by bone, tooth, pearl, coral, shell, and crustacea, certain organisms have the ability to synthesize inorganic materials with refined structures and superior physical properties that are difficult to imitate. Such biomechanisms are designated as ‘biomineralization’, which is known as an environmentally-friendly synthesis process of inorganic materials under mild conditions. Synthetic methods of generating bioinspired materials and bioceramics have been reported by many researchers [1,2,3]. Liu et al. prepared calcium carbonate (CaCO_3_)-regulated silk fibroin and estimated the drug release of doxorubicin using its vaterite microspheres [4]. DNA-Cu_3_(PO_4_)_2_ hybrid nanoflowers were synthesized by Wu et al., and these materials are predicted to employ microRNA detection as captors [5]. He et al. described the synthesis of hematite mesocrystals with hierarchical structures via collagen templates [6]. Biominerals and bioinspired materials continue to be developed and are utilized within a variety of biosensing and biomedical applications.

Even in biomineralization, organic molecules play a crucial role in the formation of the crystalline nucleus and control of crystal polymorphism in vivo, as well as crystal growth and the shaping of the whole inorganic mineral. For example, peptides, which have unique well-ordered structures within their side chains, have been used as organic molecules to synthesize inorganic materials in a variety of methods [7,8,9,10,11]. Wada et al. controlled CaCO_3_ crystallization within hydrogels by mixing polylysine and polyaspartic acid via double-diffusion methods; furthermore, the influence of peptide in the formation of the composites was elucidated [12]. “End-tethered poly(l-lysine)” monolayer brushes have been employed on silica film mineralization as reported by Wu and colleagues [13]. In summary, small amounts of peptides can affect materials morphologies and surface potentials [14].

Recently, self-assembling peptide-templated inorganic materials have been developed to readily manipulate the functional groups and secondary structures in peptides [15,16,17,18,19,20,21,22,23,24]. Lu et al. designed β-folded glutamic acid (leucine–glutamic acid)_9_ (E(LE)_9_) peptides, with its peptides working as a key player in the formation of calcium oxalate nanosheets [25]. Xu and colleagues reported the synthesis of inorganic materials based on short peptide self-assembly designed as I_m_K_n_ (e.g., isoleucine–isoleucine–isoleucine–lysine, I_3_K), I_m_E_n_ (e.g., isoleucine–isoleucine–isoleucine–glutamic acid, I_3_E), and I_m_R_n_ (e.g., isoleucine–isoleucine–isoleucine–isoleucine–arginine–arginine, I_4_R_2_) [26,27,28,29]. Xu et al. revealed that control of morphology and size of nanostructures were observed by using a self-assembling peptide template. In addition, our group previously showed that the morphologies of inorganic materials are controlled on a self-assembling peptide template [30,31,32,33] and elucidated that the secondary structures of peptides have a great impact on the resulting particles.

Hydroxyapatite (Ca_10_(PO_4_)_6_(OH)_2_, termed HAp) composes the primary inorganic contents of human tooth and bone and is a representative of biomineralization and bioinspired materials [34,35,36,37,38,39,40]. Hadagalli et al. established mineralization of porous HAp scaffolds, in which pores are obtained using organic pore formers, such as wax, wheat flour, or milk powder, and exhibit good cytocompatibility with osteoblasts in vitro [41]. Wei et al. synthesized biomineralized microspheres as follows: an amphipathic poly(l-lactide)-poly(ethylene glycol)-poly(l-lactide) triblock copolymer was coated with gelatin, then the microspheres were immersed in simulated body fluid containing dissolved alendronate. The resulting microspheres exhibited an increased effect on osteogenesis and bone regeneration compared with that of pristine microspheres lacking alendronate [42]. However, studies on HAp mineralization using self-assembled peptide templates could provide additional useful information, and applications based on peptide-template–HAp have rarely been reported.

Our main research is producing adsorbents for biosensing and bioseparation applications, that is, the materials need capable of adsorption selectively. Notably, we investigated not only the impact of calcium phosphate mineralization on peptide templates but also protein and enzyme adsorption performances by using as-synthesized materials [43,44,45]. It revealed that the morphology of the peptide–HAp hybrid materials included carboxyl groups was influenced from the secondary structures in peptides, and peptide–HAp composites with amino groups carried out for the application as glucose sensors because of its highly selective adsorption ability for proteins. In addition, we previously reported silica biomineralization on self-assembled peptide template using (leucine–lysine–leucine–leucine)_5_-PEG_70_ and (valine–lysine–valine–valine)_5_-PEG_70_ [46]. From these, we aimed at the preparation of selective protein adsorption agents using self-assembled peptide templates on HAp mineralization.

**Scheme 1:**
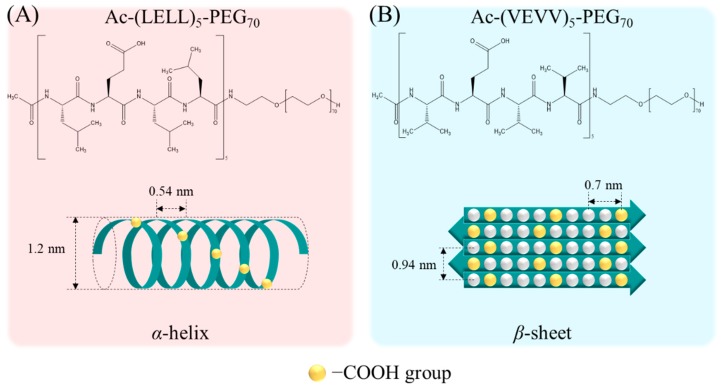
Structural formula of the peptides. (**A**) Ac-(LELL)_5_-PEG_70_ and (**B**) Ac-(VEVV)_5_-PEG_70_ were self-assembled to α-helix and β-sheet conformations, respectively.

Hence, we prepared a peptide–poly(ethylene glycol) (peptide–PEG) block copolymer by solid-phase peptide synthesis using leucine (L), glutamic acid (E), and valine (V) as rich carboxyl groups within peptide side chains (Ac-(LELL)_5_-PEG_70_ and Ac-(VEVV)_5_-PEG_70_, Scheme 1). Subsequently, calcium phosphate mineralization using well-arranged peptide templates was attempted. The aim of this study is to provide insight into the influence of peptides on hybrid particle materials and the effect(s) of protein adsorption behavior on particles.

## 2. Results and Discussion

### 2.1. Peptide–HAp Characterization

**Figure 1:**
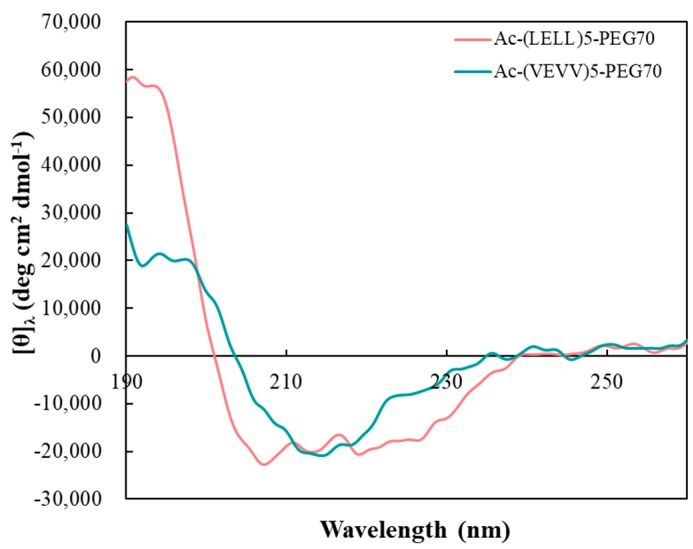
CD spectra of Ac-(LELL)_5_-PEG_70_ and Ac-(VEVV)_5_-PEG_70_. Each peptide was dissolved in 10 mM phosphate buffer (pH 7.0) and the concentration of peptide was 1.0 × 10^−5^ M.

The circular dichroism (CD) spectra of Ac-(LELL)_5_-PEG_70_ (abbreviated as LELL) and Ac-(VEVV)_5_-PEG_70_ (VEVV) are shown in Figure 1. Two negative peaks at 207 and 220 nm and a positive peak at 191 nm within LELL suggested α-helixes. Conversely, the CD spectra of VEVV showed a positive peak at 195 nm and a negative peak at 215 nm, indicating a β-sheet structure [47,48].

**Figure 2:**
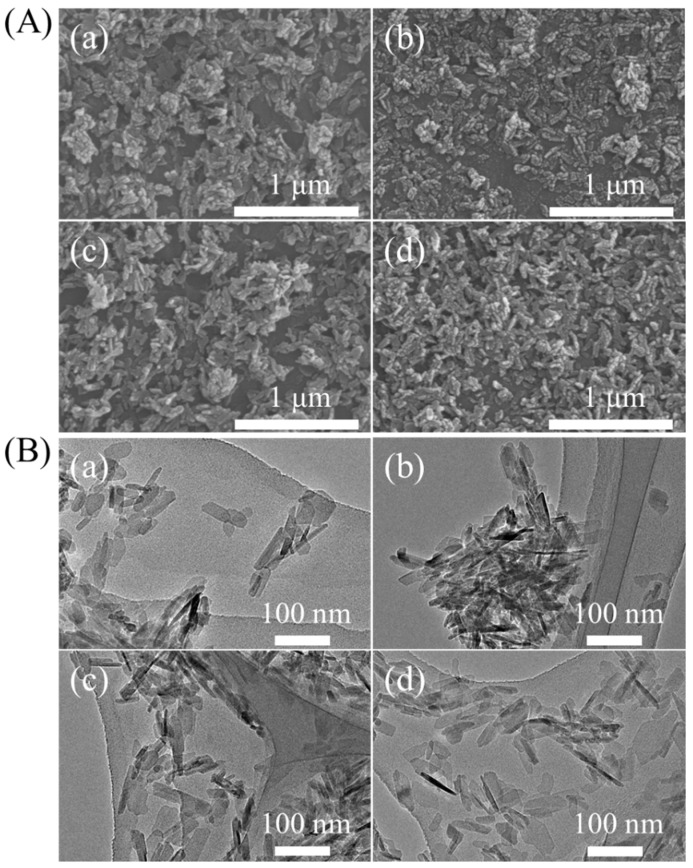
(**A**) FE-SEM and (**B**) TEM images of peptide–HAp composites: (**a**) LELL–HAp (1 mg), (**b**) LELL–HAp (3 mg), (**c**) VEVV–HAp (1 mg), and (**d**) VEVV–HAp (3 mg).

Field-emission scanning electron microscopy (FE-SEM) and transmission electron microscopy (TEM) images of LELL–HAp (1 and 3 mg) and VEVV–HAp (1 and 3 mg) were observed as shown in Figure 2A,B. SEM images of peptide–HAp display nanorods with a length of approximately 60 nm, similar to pristine HAp. Compared with LELL–HAp particles, the morphology of VEVV–HAp exhibited slightly larger plate like particles (Figure 2B).

**Figure 3:**
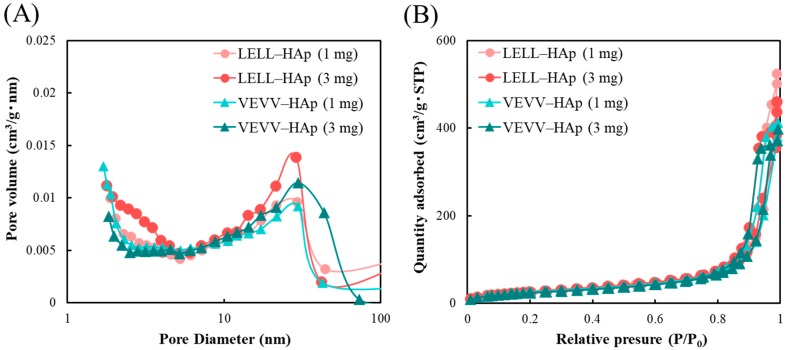
(**A**) Pore size distribution curves, and (**B**) nitrogen adsorption–desorption isotherms of LELL–HAp (1 and 3 mg) and VEVV–HAp (1 and 3 mg).

The Brunauer-Emmett-Teller (BET) surface area and pore volume and Barrett-Joyner-Halenda (BJH) pore size distribution of peptide–HAp are shown in Figure 3A,B and Table 1; nitrogen adsorption–desorption isotherms could be classified as a type IV. The specific surface areas of LELL–HAp (1 and 3 mg) and VEVV–HAp (1 and 3 mg) were found to be 106, 101, 101, and 92 m^2^ g^−1^, whereas the pore volumes were 0.81, 0.71, 0.64, and 0.62 cm^3^ g^−1^, respectively. In addition, pore sizes of 30 nm appeared in all samples. We previously observed that pore sizes of 70 nm were not present in any peptide–HAp besides bare HAp, and pore sizes of around 3 nm were confirmed in all samples. However, in the case of peptide–HAp, only α-pLys–HAp (30 and 40 mg) have pore sizes of 30 nm, of which pores may have impacted the enzyme stability of glucose oxidase immobilized on these materials [45]. As-prepared peptide–HAp is predicted to be usable for enzyme immobilization agents in biosensing and bioseparation.

The values of the Ca/P molar ratios are listed in Table 1. The Ca/P molar ratios of LELL–HAp (1 and 3 mg) and VEVV–HAp (1 and 3 mg) were 1.52, 1.50, 1.52, and 1.51, respectively. These ratios exhibited a relatively high degree of similarity to non-peptide–HAp, even though these values were lower than the stoichiometric ratio of HAp of 1.67. As a result, we observed that these calcium phosphates were low-crystallinity HAp or calcium-deficient HAp as composites of HAp and peptides.

**Figure 4:**
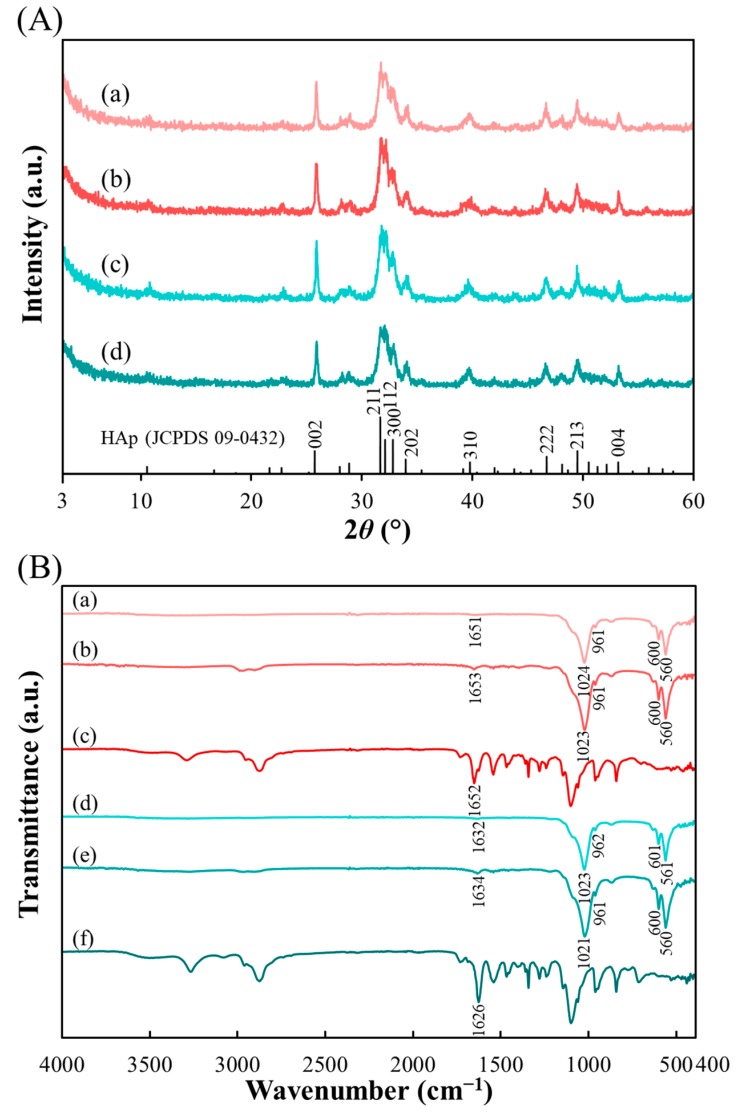
(**A**) X-ray diffraction patterns of (**a**) LELL–HAp (1 mg), (**b**) LELL–HAp (3 mg), (**c**) VEVV–HAp (1 mg), and (**d**) VEVV–HAp (3 mg) (JCPDS card no. 09-0432). (**B**) FTIR spectra of (**a**) LELL–HAp (1 mg), (**b**) LELL–HAp (3 mg), (**c**) pure Ac-(LELL)_5_-PEG_70_, (**d**) VEVV–HAp (1 mg), (**e**) VEVV–HAp (3 mg), and (**f**) pure Ac-(VEVV)_5_-PEG_70_.

Figure 4A shows the powder X-ray diffraction (XRD) patterns of peptide–HAp. The diffraction peaks at 2*θ* = 25.9°, 31.8°, 32.2°, 32.8°, 34.0°, 39.7°, 46.7°, 49.5°, and 53.2° correspond to the (002), (211), (112), (300), (202), (310), (222), (213), and (004) planes of hydroxyapatite, respectively, of which broad peaks indicate that all samples synthesized in this study were low-crystallinity HAp (JCPDS card no. 09-0432) [49].

Thermogravimetry and differential thermal analysis (TG-DTA) analysis was performed in order to confirm the peptide content in peptide–HAp composites, and peptide amounts are summarized in Table 1, which were calculated by weight losses from 200 °C to 700 °C that were attributed to peptide loss. The relative peptide amounts of LELL–HAp (1 and 3 mg) and VEVV–HAp (1 and 3 mg) were 0.43, 1.4, 0.51, and 1.5 mg, respectively. In other words, this reveals that the peptide amounts within peptide–HAp were 2.4, 7.5, 2.3, and 6.5 wt % in the particles, respectively.

The zeta potential charge of peptide–HAp was also investigated, as shown in Table 1. The surface potentials were −19.8, −12.8, −12.5, and −11.1 mV for LELL–HAp (1 and 3 mg) and VEVV–HAp (1 and 3 mg), respectively, leading to the independence of the amount of peptides.

Fourier transform infrared (FTIR) data of native peptide and peptide–HAp is shown in Figure 4B. The presence of PO_4_^3−^ functional groups in HAp can be observed by the bands at around 560, 600, 960, and 1020 cm^−1^ [49]. The −C=O stretching vibration at 1600–1700 cm^−1^ for amide I could be assessed as the peptide structure. Among these, we focused on two main peaks at around 1650 and 1630 cm^−1^, attributed to α-helix and β-sheet structure [48,50]. The LELL–HAp (1 and 3 mg) spectra had peaks corresponding to HAp; the PO_4_^3−^ bending vibration (O–P–O) at 560 and 600 cm^−1^ and the peaks at around 961 and 1024 cm^−1^ originated from bending modes of the P–O bond in PO_4_^3−^. Moreover, the characteristic of an α-helix in LELL from 1651 to 1653 cm^−1^ was observed. In the case of pure LELL, the band at 1652 cm^−1^ is attributed to an α-helix structure. The characteristic peaks at around 560, 600, 961, and 1022 cm^−1^ for VEVV–HAp (1 and 3 mg) could be designated as PO_4_^3−^ groups in HAp. Additionally, the bands registered at 1632 and 1634 cm^−1^ for VEVV–HAp (1 and 3 mg, respectively) were ascribed to β-sheet peaks. For VEVV, the same band (β-sheet structure) was observed at 1626 cm^−1^. According to these results, the presence of both HAp and peptide in peptide–HAp could be confirmed; furthermore, the peaks derived from each peptide secondary structure were also classified.

Secondary structural contents of two peptides and that bound with Ca ions were clarified by FTIR analysis (Table 2). Firstly, native LELL and VEVV had higher contents of α-helixes and β-sheets, respectively, as attributed to the CD data. Moreover, to investigate the secondary structure of peptide in peptide–HAp, we prepared each peptide bond with Ca ions as follows: 3 mg LELL or VEVV was added to 20 mL of prepared (CH_3_COO)_2_Ca solution (15 mM). After stirring for 2 h at 20 °C, the solid materials were obtained via the freeze-drying process. Most secondary structures were of an α-helix content for LELL bound with Ca ions; meanwhile, VEVV bound with Ca ions not only contained primarily β-sheet structures but also various secondary structural contents (α-helixes, β-turns, and others).

**Figure 5:**
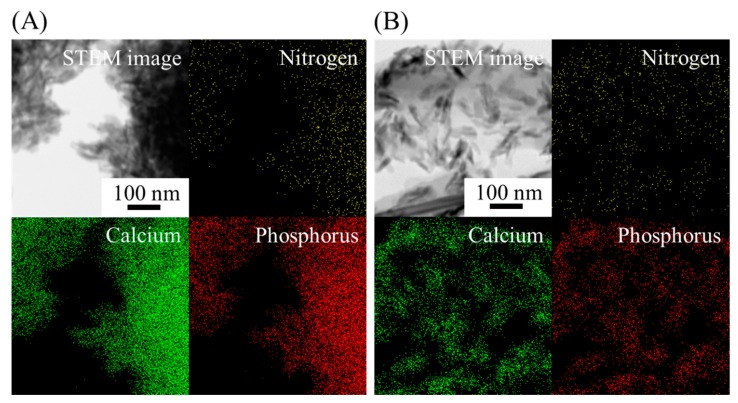
STEM images and EDX maps of elements of (**A**) LELL–HAp (3 mg) and (**B**) VEVV–HAp (3 mg). Nitrogen (yellow), calcium (green), and phosphorous (red) are displayed.

To investigate the elemental distribution in peptide–HAp particles, especially the peptide, scanning transmission electron microscopy (STEM) images, and energy-dispersive X-ray spectroscopy (EDX) maps were utilized (Figure 5A,B). Nitrogen is attributed to the peptides and calcium, and phosphorous corresponds to HAp. Nitrogen (yellow) is homogeneously distributed throughout nanoparticles; thus, it could be assumed that the peptides are distributed in the particles.

### 2.2. Protein Adsorption on Peptide–HAp

**Figure 6:**
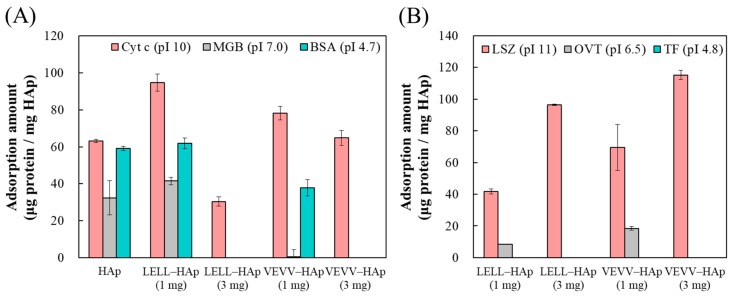
(**A**) Protein (cytochrome c, myoglobin, and bovine serum albumin) adsorption on HAp, LELL–HAp (1 and 3 mg), and VEVV–HAp (1 and 3 mg). (**B**) Protein (lysozyme, conalbumin, and transferrin) adsorption on LELL–HAp (1 and 3 mg) and VEVV–HAp (1 and 3 mg).

Three typical proteins with different isoelectric points (i.e., cytochrome c; Cyt c, myoglobin; MGB, and bovine serum albumin; BSA) were adsorbed not only on peptide–HAp but also non-peptide–HAp (Figure 6A). The capacity and tendency for protein adsorption on HAp were similar to the results obtained in a previous report [43]. The amounts of adsorbed Cyt c on LELL–HAp (1 mg) and VEVV–HAp (1 mg) were 94.8 and 78.3 μg mg^−1^, respectively. In addition, the adsorbed MGB amounts were 41.5 and 0.571 μg mg^−1^ for LELL–HAp (1 mg) and VEVV–HAp (1 mg), and the BSA capacities were 61.8 and 37.9 μg mg^−1^, respectively. In terms of the amounts of adsorbed Cyt c for LELL–HAp (3 mg) and VEVV–HAp (3 mg), the capacities were 30.4 and 64.8 μg mg^−1^, respectively, whereas two samples had either no or extremely low adsorption amounts for MGB and BSA. Furthermore, lysozyme (LSZ), conalbumin (ovotransferrin; OVT), and transferrin (TF) was adsorbed on peptide–HAp to confirm its selectivity only for basic protein (Figure 6B). The amounts of adsorbed LSZ on LELL–HAp (1 mg), VEVV–HAp (1 mg), LELL–HAp (3 mg), and VEVV–HAp (3 mg) were 41.9, 69.6, 96.4, and 115 μg mg^−1^, respectively. For all peptide–HAp, adsorption amounts of OVT and TF were either no or extremely low. From these results, it could be hypothesized that selectivity for protein adsorption on peptide–HAp is due to the presence of glutamic acid (E) within the peptides. Moreover, VEVV–HAp exhibited a high blocking effect itself for the other proteins during the maintenance of Cyt c and LSZ adsorption capacities with increasing peptide amounts.

### 2.3. Carboxyl Group Density in Peptide–HAp

The determination of the carboxyl group density in peptide–HAp indicated the cause for selectivity of protein adsorption on peptide–HAp. The densities of carboxyl groups were 2.33, 3.38, 5.97, and 13.0 nmol m^−2^ for LELL–HAp (1 mg), VEVV–HAp (1 mg), LELL–HAp (3 mg), and VEVV–HAp (3 mg), respectively, in which the density of peptide–HAp was improved with increasing amounts of peptide. Moreover, the density of VEVV–HAp (3 mg) was more than twofold higher compared with LELL–HAp (3 mg). From the results, the difference in Cyt c adsorption amounts between VEVV–HAp (3 mg) and LELL–HAp (3 mg) could be explained by the high carboxyl group density in VEVV–HAp (3 mg).

## 3. Materials and Methods

### 3.1. Materials

All chemicals were of analytical grade and were used as received without further purification. Calcium acetate monohydrate [(CH_3_COO)_2_Ca·H_2_O] and diammonium hydrogen phosphate [(NH_4_)_2_HPO_4_] were obtained from FUJIFILM Wako Pure Chemical Co. (Osaka, Japan). Cytochrome c from equine heart [Cyt c; isoelectric point (pI) = 10, molecular weight (Mw) = 12,300 Da], myoglobin from equine skeletal muscle (MGB; pI = 7.0, Mw = 17,800 Da), bovine serum albumin (BSA; pI = 4.7, Mw = 67,000 Da), lysozyme from chicken egg white (LSZ; pI = 11, Mw = 14,300 Da), conalbumin from chicken egg white (OVT; pI = 6.5, Mw = 76,000 Da), and transferrin human (TF; pI = 4.8, Mw = 80,000 Da) were purchased from Merck KGaA (Darmstadt, Germany). 1-Ethyl-3-(3-dimethylaminopropyl)carbodiimide hydrochloride (EDC; Mw = 191.7), *N*-hydroxysuccinimide (NHS; Mw = 115.1), and 5-aminofluorescein (Mw = 347.3) were obtained from Tokyo Kasei Kogyo Co. (Tokyo, Japan). The Bio-Rad protein assay dye reagent concentrate was purchased from Bio-Rad Laboratories (Hercules, CA, USA).

### 3.2. Preparation of Peptide–HAp Particles

Two peptides (Ac-(LELL)_5_-PEG_70_ and Ac-(VEVV)_5_-PEG_70_) were prepared via solid-phase peptide synthesis according to our previous reports [30,31,32,33]. One or 3 mg of Ac-(LELL)_5_-PEG_70_ or Ac-(VEVV)_5_-PEG_70_ was added to a 20 mL solution of dissolved (CH_3_COO)_2_Ca (52.8 mg), followed by stirring for 30 min at 20 °C. After addition of 20 mL (NH_4_)_2_HPO_4_ solution (23.8 mg), the mixture was heated to 60 °C at a heating rate of 1 °C min^−1^. The temperature was maintained for 3 h with stirring, and then the precipitant was collected by centrifugation at 6000 rpm for 10 min. The final products were washed twice with deionized water (Milli-Q, Merck KGaA, Darmstadt, Germany) and freeze-dried. To compare protein adsorption behavior, non-peptide–HAp was also synthesized using the same process as peptide–HAp.

### 3.3. Characterization of Synthesized Peptide–HAp

To analyze the secondary structure of Ac-(LELL)_5_-PEG_70_ and Ac-(VEVV)_5_-PEG_70_, the CD spectrum was measured using J-820 (JASCO Co., Tokyo, Japan) in a range scanning of 190–260 nm. The morphologies of all peptide–HAp samples were visualized with FE-SEM (S-4300, Hitachi Ltd., Tokyo, Japan) under 10.0 kV accelerating voltage and TEM (JEM-2010, JEOL Ltd., Tokyo, Japan) at 200 kV accelerator voltage. The specific surface area, pore volume, and pore size distribution were calculated on the basis of nitrogen adsorption–desorption isotherms using a TriStar 3000 (Shimadzu Co., Kyoto, Japan) via the BET and BJH models. For the measurement of the Ca/P molar ratio of synthesized peptide–HAp, inductively coupled plasma optical emission spectrometry (ICP-OES; IRIS Advantage, Thermo Fisher Scientific Inc., Waltham, MA, USA) was employed. The calculated peptide amounts of peptide–HAp were analyzed by TG-DTA (Thermo Plus TG 8120, Rigaku Co., Tokyo, Japan) in the operation range of room temperature to 1000 °C (heating rate of 10 °C min^−1^). ELSZ-1000 (Otsuka Electronics Co., Tokyo, Japan) was employed to measure the zeta potential of peptide–HAp, with the samples prepared via dispersion in 10 mM phosphate buffer of pH 7.0 with sonication for 3 min. XRD (SmartLab SE/B1, Rigaku Co., Tokyo, Japan) analysis was carried out using CuKα radiation operated at an accelerator voltage of 40 kV and a beam intensity of 30 mA. The XRD patterns were collected at a step size of 2.0° min^−1^ and a 2*θ* range between 3° and 60°. FTIR spectra in the 400–4000 cm^−1^ range were recorded by FT/IR-4700 (JASCO Co., Tokyo, Japan) with attenuated total reflection. STEM (JEM-2100 Plus, JEOL Ltd., Tokyo, Japan) operated at 200 kV with EDX (Noran System 7, Thermo Fisher Scientific Inc., Waltham, MA, USA) was used to analyze the element distribution of peptide over peptide–HAp.

### 3.4. Protein Adsorption on Peptide–HAp

Each protein (i.e., Cyt c, MGB, BSA, LSZ, OVT, and TF) was dissolved in 10 mM phosphate buffer (pH 7.0), and the protein solution was prepared at a concentration of 250 μg mL^−1^. Peptide–HAp (1.5 mg) was mixed with a 1 mL protein solution, followed by stirring overnight at 20 °C. The supernatant was separated from the mixture by centrifugation at 14,000 rpm for 5 min, and excess protein in the supernatant was estimated using the Bradford method by UV–Vis spectroscopy (Infinite F200 PRO, Tecan Group Ltd., Mӓnnedorf, Switzerland) at λ = 595 nm. Bio-Rad protein assay dye was employed for the evaluation of protein adsorption performance with the equation

(1) $$Q=Q_{0}\left(\frac{I_{0}-I}{I_{0}}\right)$$

where *Q* is the adsorption capacity of protein on peptide–HAp, *Q*_0_ is the initial amount of protein, *I*_0_ is the initial absorbance intensity in the supernatant, and *I* is the absorbance intensity of the supernatant following adsorption.

### 3.5. Calculation of Carboxyl Group Density in Peptide–HAp

First, EDC (42.9 mg) and NHS (5.1 mg) were each dissolved in 3 mL of 10 mM phosphate buffer (pH 7.0), and the solutions (500 μL) were mixed together. One mg of peptide–HAp was added to the mixture and stirred for 3 h at 20 °C. The solid materials were separated by centrifugation at 14,000 rpm for 5 min and then washed three times with 10 mM phosphate buffer (pH 7.0). The precipitant was resuspended in the same phosphate buffer (500 μL), and then 500 μL of 5-aminofluorescein solution (8 μg mL^−1^) was added to the suspension. After stirring in the dark overnight at 20 °C, the solid materials were collected by centrifugation at 14,000 rpm for 5 min and washed with the aforementioned buffer. Finally, the carboxyl group density in the precipitant was redispersed in a 1 mL buffer and measured using a spectrofluorophotometer (RF-5300PC, Shimadzu Co., Kyoto, Japan), of which the excitation and emission wavelengths were 494 and 521 nm, respectively.

## 4. Conclusions

In summary, we designed two types of self-assembling peptides with different secondary structures: (leucine–glutamic acid–leucine–leucine)_5_-PEG_70_ (LELL) and (valine–glutamic acid–valine–valine)_5_-PEG_70_ (VEVV), and these peptides were used as templates for HAp biomineralization. Moreover, we also investigated the effect of secondary structures within peptide-template–HAp on the particles and protein adsorption behavior. It could be shown that as-synthesized peptide LELL or VEVV showed almost entirely α-helix or β-sheet contents within secondary structures, respectively. The morphologies of all peptide–HAp were similar to bare HAp, whereas VEVV–HAp displayed a slightly plate-like structure. Additionally, all peptide–HAp have pore sizes of 30 nm, which may be expected for enzyme stability on enzyme immobilization, as indicated in our previous study. Furthermore, for the adsorption properties of proteins, it was revealed that each peptide–HAp specifically adsorbed basic protein (i.e., Cyt c and LSZ). With increasing amounts of peptide, the blocking effects for proteins, except for basic protein, were also increased. Overall, the reason that VEVV–HAp (3 mg) with β-sheet structures exhibited increased Cyt c adsorption amounts compared with LELL–HAp (3 mg) containing α-helix structures is explained as follows: the carboxyl group density at the surfaces of VEVV–HAp (3 mg) was more than 2-times higher compared with LELL–HAp (3 mg) while the carboxyl group density of peptide–HAp incorporated 1 mg of peptide amount was lower than that of peptide–HAp (3 mg). From these results, it can be stated that synthesized HAp on a self-assembling peptide template could be useful as a carrier for protein immobilization in biosensing and bioseparation applications and as enzyme-stabilizing agents.

## Figures and Tables

**Table 1:** Structural properties, Ca/P molar ratio, amount of peptide, and zeta potential of peptide–HAp composites.

Sample	Surface Area ^(a)^ (m^2^ g^−1^)	Pore Volume ^(a)^ (cm^3^ g^−1^)	Ca/P Molar Ratio ^(b)^	Amount of Peptide ^(c)^ (mg)	Zeta Potential ^(d)^ (mV)
LELL–HAp (1 mg)	106	0.81	1.52	0.43	−19.8
LELL–HAp (3 mg)	101	0.71	1.50	1.4	−12.8
VEVV–HAp (1 mg)	101	0.64	1.52	0.51	−12.5
VEVV–HAp (3 mg)	92	0.62	1.51	1.5	−11.1

^(a)^ The specific surface area, pore volume, and pore size distribution of peptide–HAp were calculated on the basis of nitrogen adsorption–desorption isotherms by the Brunauer-Emmett-Teller (BET) and Barrett-Joyner-Halenda (BJH) methods. ^(b)^ To measure the Ca/P molar ratio of peptide–HAp, inductively coupled plasma optical emission spectrometry (ICP-OES) was employed. ^(c)^ Peptide amounts within composites were determined by thermogravimetry and differential thermal analysis (TG-DTA). ^(d)^ The surface potential of peptide–HAp was measured via electrophoretic light scattering methods, whereas the particles were prepared by dispersion in 10 mM phosphate buffer with pH 7.0 and sonication for 3 min.

**Table 2:** Secondary structures (%) of Ac-(LELL)_5_-PEG_70_ and Ac-(VEVV)_5_-PEG_70_.

Sample	α-Helix	β-Sheet	β-Turn	Other
LELL	94%	— *	3%	3%
LELL + Ca ^(a)^	>99%	— *	— *	— *
VEVV	— *	96%	1%	3%
VEVV + Ca ^(a)^	2%	47%	24%	27%

* Trace percent. ^(a)^ Each peptide (Ac-(LELL)_5_-PEG_70_ and Ac-(VEVV)_5_-PEG_70_) (3 mg) was mixed with 20 mL (CH_3_COO)_2_Ca solution (15 mM) and stirred for 2 h at 20 °C. The resulting product was then freeze-dried.

## Abbreviations

HAp	Hydroxyapatite
PEG	Poly(ethylene glycol)
CD	Circular dichroism
FE-SEM	Field-emission scanning electron microscopy
TEM	Transmission electron microscopy
BET	Brunauer-Emmett-Teller
BJH	Barrett-Joyner-Halenda
XRD	Powder X-ray diffraction
TG-DTA	Thermogravimetry and differential thermal analysis
FTIR	Fourier transform infrared
STEM	Scanning transmission electron microscopy
EDX	Energy-dispersive X-ray spectroscopy
Cyt c	Cytochrome c
MGB	Myoglobin
BSA	Bovine serum albumin
LSZ	Lysozyme
OVT	Conalbumin
TF	Transferrin
EDC	1-Ethyl-3-(3-dimethylaminopropyl)carbodiimide hydrochloride
NHS	*N*-Hydroxysuccinimide
